# Near Real-Time Detection of *E. coli* in Reclaimed Water

**DOI:** 10.3390/s18072303

**Published:** 2018-07-16

**Authors:** Samendra Sherchan, Syreeta Miles, Luisa Ikner, Hye-Weon Yu, Shane A. Snyder, Ian L. Pepper

**Affiliations:** 1 Department of Global Environmental Health Sciences, Tulane University, 1440 Canal St, New Orleans, LA 70112, USA; 2 Water & Energy Sustainable Technology (WEST) Center, The University of Arizona, 2959 W. Calle Agua Nueva, Tucson, AZ 85745, USA; syrt8miles@gmail.com (S.M.); ikner@email.arizona.edu (L.I.); hyeweonyu@gmail.com (H.-W.Y.); ssnyder@ntu.edu.sg (S.A.S.); ipepper@ag.arizona.edu (I.L.P.); 3 Public Health Laboratory, San Diego County Health and Human Services Agency, San Diego, CA 92101, USA; 4 Nanyang Environment & Water Research Institute (NEWRI), Nanyang Technological University (NTU), 50 Nanyang Avenue, Singapore 639798, Singapore

**Keywords:** water quality, online sensors, microorganism, water supply, monitoring, water reuse

## Abstract

Advanced treatment of reclaimed water prior to potable reuse normally results in the inactivation of bacterial populations, however, incremental treatment failure can result in bacteria, including pathogens, remaining viable. Therefore, potential microorganisms need to be detected in real-time to preclude potential adverse human health effects. Real-time detection of microbes presents unique problems which are dependent on the water quality of the test water, including parameters such as particulate content and turbidity, and natural organic matter content. In addition, microbes are unusual in that: (i) viability and culturability are not always synonymous; (ii) viability in water can be reduced by osmotic stress; and (iii) bacteria can invoke repair mechanisms in response to UV disinfection resulting in regrowth of bacterial populations. All these issues related to bacteria affect the efficacy of real-time detection for bacteria. Here we evaluate three different sensors suitable for specific water qualities. The sensor A is an on-line, real-time sensor that allows for the continuous monitoring of particulates (including microbial contaminants) using multi-angle-light scattering (MALS) technology. The sensor B is a microbial detection system that uses optical technique, Mie light scattering, for particle sizing and fluorescence emission for viable bacteria detection. The last sensor C was based on adenosine triphosphate (ATP) production. *E. coli* was used a model organism and out of all tested sensors, we found the sensor C to be the most accurate. It has a great potential as a surrogate parameter for microbial loads in test waters and be useful for process control in treatment trains.

## 1. Introduction

As water quality perturbations related to rapid population growth and polluted freshwater resources continue to increase throughout the world, effective potable water quality monitoring and advanced treatment have become critical for the water industry. Also, due to extreme water scarcity/water stress, direct potable reuse (DPR) is now being considered to boost supplies of drinking water. However, a major concern in safeguarding water purity is the real-time detection of microbial contaminants. Currently there is no available technology that will specifically identify a microorganism in real-time. Thus, it is critical to investigate the operation and performance of some commercially available online sensor technologies to ensure that they are in fact suitable for the intended use. This information will be essential to broaden our understanding of the role of early warning systems to monitor microbial contaminants in real-time.

Bacteria are prokaryotic microorganisms that are the least complex of living microbes but offer the greatest metabolic flexibility and diversity. Bacteria can thrive within a variety of environments, including potable water distribution systems [1]. Despite deliberate disinfection of water via chlorine by utilities, almost all potable water within distribution systems can still contain vast populations of bacteria of up to 10^6^ culturable cells per mL. Most of these bacteria pose no threat to human health, and function as living ecosystems within the distribution system [1], however, pathogenic bacteria can also be found within potable water because of intrusion events due to accidental pipe breakage, or perhaps even deliberate contamination via terrorist activities. More recently, enhanced national and international water shortages have resulted in reclaimed water being utilized for potable reuse. Prior to potable reuse, the reclaimed water must be subjected to advanced treatment to remove both chemical contaminants and potential microbial pathogens. For example, UV advanced oxidation is known to be effective for the inactivation of viruses, bacteria and protozoa [2], and the degradation of organic micro- pollutants [3]. However, all advanced treatment options, including advanced oxidation and membrane filtration, can be subject to incremental failure that can result in progressively less efficient removal of contaminants. For all these reasons, to safeguard potable water and reduce consumer vulnerability, on-line sensors must be utilized for real-time detection of both chemical and microbial contaminants.

**Figure 1:**
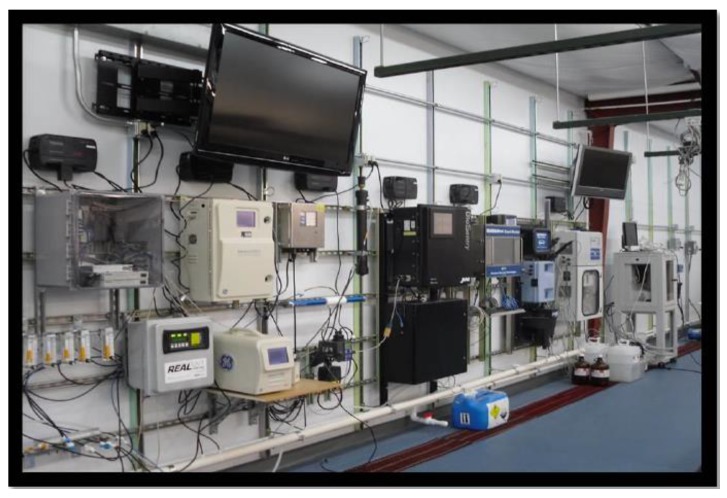
Real-time Sensor Laboratory.

Real-time detection of bacteria poses several challenges that make their detection in water potentially more difficult than the detection of chemicals. First, while many viable pathogens can be readily cultured using standard microbiological techniques, there are also many other pathogens that are viable and metabolically active, but cannot be cultured in vitro—the so-called viable-but-non-culturable (VBNC) bacteria [2]. Second, although ultraviolet disinfection is known to inactivate pathogens [3], many bacterial populations are capable of activating nucleic acid repair mechanisms and proliferating once again to appreciable numbers. Fecal coliforms and *E. coli* can also regrow in environmental samples [4,5]. Finally, it should be noted that bacterial pathogens in water are almost universally accompanied by large populations of non-pathogenic microbes, enhancing the difficulty of their detection. All these issues need to be addressed to ensure complete elimination of bacterial contaminants in water. At the University of Arizona, we have developed a Real-Time Sensor Laboratory for water which is currently housed in the newly completed Water and Energy Sustainable Technology Center (WEST, Figure 1). Over the past 5 years we have evaluated and enhanced the efficacy of both chemical and microbial real-time sensors and assays utilized to detect contaminants in water. In this study, our objective is to evaluate commercially available sensors to monitor *E. coli* in real time using different water sources.

## 2. Methods

We conducted laboratory and field-scale evaluations of multiple real-time detection strategies for the detection of bacterial contaminants in a variety of water quality matrices. Specifically, the detection evaluations were initiated using distilled water, followed by tap water and finally secondary-treated wastewater effluents. Initial tests were conducted by spiking waters with known concentrations of bacteria into fixed water volumes. Subsequent evaluations focused on the detection of natural, existing populations within secondary-treated effluent waters resulting from wastewater treatment plants.

### 2.1. Multi-Angle Light Scattering Technology (Sensor A)

This sensor is an on-line, real-time sensor that allows for the continuous monitoring of particulates (including microbial contaminants) using multi-angle-light scattering (MALS) technology. The sensor contains a laser beam that strikes individual cells or particles in water, resulting in unique light scattering patterns that depend on the size and morphological characteristics of the target particle. Data obtained are compared to patterns within a computerized database which are subsequently categorized as rods, spores, protozoa, or as unknown entities. The data output is shown in counts per minute for each category, and can be converted to organisms/mL, allowing for comparison with culturable counts (cfu/mL).

We used MALS for the detection of *E. coli* in distilled and tap water. For all experiments, a baseline output from the sensor was established for 30–60 min by continuous passage of either DI or tap water. Subsequently, the sensor was challenged with *E. coli* (#15597, ATCC, Manassas, VA, USA) that was cultured until the late log growth phase was reached using tryptic soy broth (TSB, Cat. #211825, Becton Dickinson, Franklin Lakes, NJ, USA). The culture was pelleted by centrifugation (4000 rpm for 25 min) and re-suspended in fresh phosphate-buffered saline (PBS); the process of centrifugation and re-suspension was conducted three times. The *E. coli* suspension was then diluted into a 50 L carboy containing 45 L of distilled or tap water to achieve a target concentration of ≃ 10^3^, 10^4^, 10^5^, or 10^6^ cfu/mL. Prior to the start of each experiment, the 45 L water sample was mixed with a water pump for 5 min. The experiment was initiated by pumping the water sample through the distribution system, and through the in-line sensor. During the injection, critical parameters including pressure, temperature, and water flow rate (1.2 L/min for 30 min) were maintained to create a steady state injection. Fifteen milliliter water samples collected at the 10, 15, 20, 25, and 30-min time points were serially diluted and plated onto tryptic soy agar (TSA, Cat. #236920, Becton Dickinson, Franklin Lakes, NJ, USA). The plates were incubated for 24 h at 37 °C, and colonies were counted to determine the culturable bacterial concentration in the water samples. The samples were also stained with acridine orange to determine total direct cell counts by acridine orange direct counts (AODC) [6]. Total counts and culturable counts were compared to the sensor output.

Because the MALS principle of detection relies on a laser beam striking particulates in water, experiments were conducted to determine if this sensor could distinguish between colloidal (inert) particulates and biological (bacterial) particulates. Fixed volumes (0.5, 2.5, 5, 7, 10 and 15 mL) of a 4000 nephelometric turbidity units (NTU) turbidity standard (Cat. #8830-32, Ricca Chemical, Arlington, TX, USA) were added to 45 L of DI water and injected through the system for 20 min to evaluate the sensor response to turbidity.

### 2.2. Real-Time Detection via Fluorescence Emission (Sensor B)

This sensor is a microbial detection system that uses optical techniques such as Mie light scattering for particle sizing and fluorescence emission for viable bacteria detection. First, it classifies the microbial size of particles (0.3 to 15 µm in spherical diameter) in water by Mie light scattering analysis with photodiode, and the particles are counted as an inert particle (cells/mL). After gating the inert particles, the instrument interrogates each particle with laser illumination, which excites intrinsic fluorescence of bacterial cells as universal “bio-marker” metabolites to differentiate viable bacteria from inert particles. The unique and distinguishable fluorescence emission from the metabolites (i.e., NADH and riboflavin) produced by bacteria and fungi is detected and counted as bio cells (cells/mL). Thus, this sensor gives counts of inert particles and viable biological cells. In contrast to traditional microbial methods, this sensor offers a continuous sampling mode with no reagents or labor required.

#### 2.2.1. Real-Time Monitoring of *E. coli* in Distilled and Tap Water

For all experiments, a baseline output from this sensor was established for 30 to 60 min by continuous passage of either DI or tap water. *E. coli* 15597 was grown in Tryptic Soy broth (Becton Dickinson, Franklin Lakes, NJ, USA) to late log phase. For one set of experiments, the cells were centrifuged (4000 rpm for 25 min), and then suspended in fresh PBS to remove organic growth medium components; the centrifugation-washing process was performed three times. The washed *E. coli* suspension was diluted into a 50 L carboy containing 45 L of water to achieve a final concentration of 10^1^, 10^2^, 10^3^, 10^4^, 10^5^, or 10^6^ cfu/mL. The 45 L water sample was mixed with a water pump for 5 min, and the experiment was initiated by pumping the seeded water sample through the in-line sensor. During the injection, the pressure and temperature remained constant with a water flow rate of 1.2 L/min for 30 min to create a steady state injection. Fifteen milliliter water samples were obtained at the 10, 15, 20, 25, and 30 min time points, and were subsequently diluted and plated on TSA (BD). The plates were incubated for 24 h at 37 °C, and colonies were counted to confirm the cultural bacterial concentration in the water samples.

#### 2.2.2. Real-Time Detection of *E. coli* in Modelled Reclaimed Water

*E. coli* 15597 grown to late log phase in TSB (BD) was washed and freshly prepared by centrifugation (4000 rpm for 25 min) and resuspension in PBS to remove organic matters in growth medium. The washed *E. coli* suspension was serially diluted in DI water to make a final concentration of 0, 1, 10^1^, 10^2^, 10^3^, 10^4^, 10^5^, 10^6^, and 10^7^ cfu/mL. To evaluate the signal interference by natural organic matter (NOM) in water, Suwannee River NOM (Cat no. 2R101N, International Humic Substances Society, St. Paul, MN, USA) was spiked at the concentrations of 0, 0.1, 1, 2.5, and 5 mg/L. The bacterial suspension flowed through this sensor at 30 mL/min, and the output data from the sensor were recorded in real-time and processed to calculate the average of the stable signal.

The samples were collected to perform absorption and fluorescence excitation-emission matrix (FL-EEM), which were analyzed using Aqualog fluorometer (Horiba, Kyoto, Japan) equipped with a 1 cm path length quartz cuvette. To obtain FL-EEM, excitation wavelengths were incrementally increased from 220 to 450 nm, and the emitted fluorescence was collected at the wavelengths of 250–580 nm. Data processing was conducted using MATLAB (The Mathworks Inc., Natick, MA, USA), which includes corrections for Raleigh/Raman scattering and inner filter effects (i.e., the absorbance of water matrix) [6].

### 2.3. Real-Time Detection via ATP Production (Sensor C)

Because of the issues related to the detection of bacteria via on-line sensors, a different approach to the real-time detection of bacteria in water was utilized—namely adenosine triphosphate (ATP) production. This assay is a rapid, non-specific measure of total microbial content in water based on adenosine triphosphate (ATP) production. ATP is a molecule found only in and around living cells. ATP is measured in Relative Light Units (RLU), which may be converted into Microbial Equivalent values based on the average bacterial (*E. coli)* cell ATP level of 1 femtogram (fg). The three main advantages of the test assay are: (i) Real-time feedback (<5 min); (ii) Measurement of culturable and VBNC microorganisms (nearly 100% of microbes detected); and (iii) Field-ready (Portable).

A Monitor Biological Growth in Water assay was utilized to determine the microbial load in two secondary effluents obtained from two wastewater treatment plants (WWTPs). In addition, tests were conducted on the permeate and brine produced by Reverse Osmosis (RO) of the effluent feed water. The microbial equivalent counts obtained were compared to culturable heterotrophic plate counts obtained by dilution and plating on R2A media.

Cellular ATP (cATP) represents the amount of ATP contained within living cells and is a direct indication of total living biomass quantity as depicted by Equation (1):

(1) $$cATP\left(pg\frac{ATP}{mL}\right)=\frac{RLU_{cATP}}{RLU_{ATP1}}\times 10,000\frac{\left[pg\;ATP\right]}{V_{sample}\left[mL\right]}$$

*NOTE*: When applicable, subtract RLU_bg_ from RLU_cATP_ prior to executing the above calculation.

To communicate results on the same basis as traditional culture tests. cATP results are converted into Microbial Equivalents (ME’s). This is based on the established conversion that 1 *E. coli*-sized bacteria contains 0.001 pg (1 ft) of ATP as shown in Equation (2):

(2) $$cATP\left(\frac{ME}{mL}\right)=cATP\left(\frac{pg\;ATP}{mL}\right)\times \frac{1\;ME}{0.001\;pg\;ATP}$$

### 2.4. Data Analysis

Statistical analyses were performed using R software and MS-Excel. The relationship between sensor outputs and cell counts were significant where *p* < 0.05.

## 3. Results and Discussion

### 3.1. Multi-Angle Light Scattering Technology [Sensor A]

**Figure 2:**
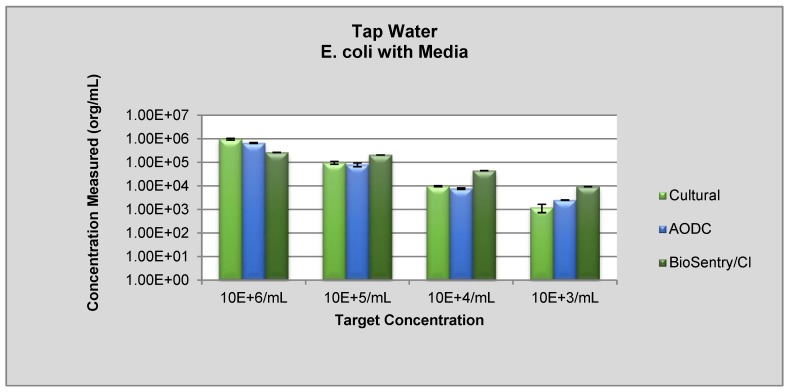
The sensor A response to *E. coli* in tap water.

Data show that this on-line sensor can detect the injected *E. coli* over a range of 10^3^–10^6^ cfu/mL in either DI or tap water (Figure 2). Sensor counts obtained from spiked tap water tended to be higher than those obtained from DI water, most likely due to the indigenous bacteria already present in tap water. These data also indicate that the sensor counts correlated closely with counts obtained via culture or the Acridine Orange Direct Counts (AODC). Thus, the sensor counts obtained in real-time offer obvious advantages over the other two assays which require 4 to 6 h to conduct.

However, particulates in water were shown to interfere with the efficacy of the sensor. When known turbidity standards were added to the test water, this sensor was unable to distinguish between the added inert particulates and the biological *E. coli* cells, resulting in an overestimation of the number of biological cells in the water. Specifically, the addition of a turbidity suspension of 0.3 NTU resulted in a 38% increase in the sensor output. A second disadvantage of the sensor is that it does not distinguish between live and dead cells, since the assay only relies on the laser beam striking a cell not the metabolic state of the cell [6,7,8,9]. Thus, this sensor gives no indication of the viability nor the potential public health hazard of the detected cells. This on-line sensor can detect biological cells in water in real time. Disadvantages include lack of effectiveness in turbid waters and the inability to distinguish between live and dead cells. However, the sensor could be used for process control of water treatment since an increase in particulate counts could indicate treatment failure

### 3.2. Real-Time Detection via Fluorescence Emission [Sensor B]

**Figure 3:**
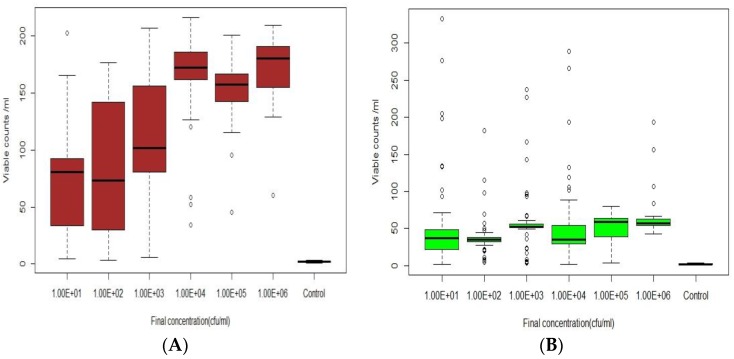
The sensor B response to *E. coli* spiked in Deionized (DI water: **A**) With Tryptic soy Broth (TSB) growth media (dirty) and (**B**) Washed cells (clean).

**Figure 4:**
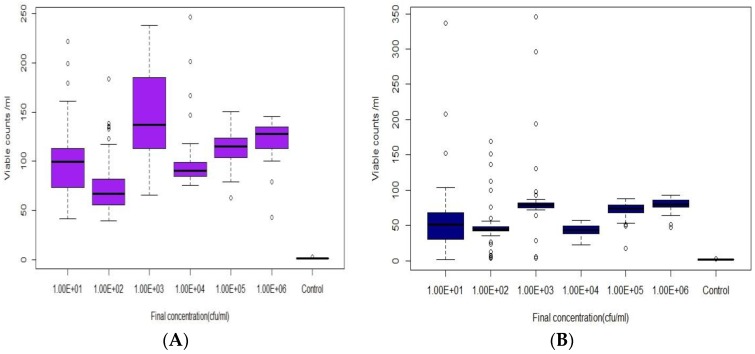
The sensor B response to *E. coli* spiked in Filtered tap water: (**A**) with TSB growth media (dirty) and (**B**) Washed cells (clean).

Experiments were performed by inoculating DI and tap water with *E. coli* suspended in either growth media or cleaned (cells removed from media and washed) at concentrations between 10 and 10^6^ cfu/mL. Samples were injected into a field scale test-bed distribution system (DS) for 30 min to achieve a steady state of the target concentration. Sensor responses to *E. coli* intrusion of DI water or filtered tap water (1 µm) are shown as boxplots (Figure 3 and Figure 4). Figure 3A shows that unwashed injections of *E. coli* in DI water induced significant sensor responses over a range of 10^1^ to 10^6^ cfu/mL. However, a statistical ANOVA and Turkey HSD test showed that there was no significant difference (*p* = 0.54) between the sensor responses to 10 and 100 cfu/mL. In addition, the sensor response to increased concentrations of *E. coli* was not linear since there was no significant difference in the responses to 10^4^, 10^5^, or 10^6^ cfu/mL; this was likely due to saturation at these higher concentrations. Most importantly, there was a significant difference (*p* < 0.05) between control (no *E. coli*) and concentrations between 10 cfu/mL and 10^6^ cfu/mL responses, indicating that low *E. coli* intrusion events could be successfully detected by this sensor. When washed cells were intruded, the sensor responses at all cell concentrations were lower than from corresponding dirty injections (Figure 3B and Figure 4B). These data imply that there were fewer viable cells in the water when no growth media was added, perhaps due to cell death following osmotic shock after introduction into the water. Similar trends for clean and dirty injections of filtered tap water were observed. Significant differences (*p* < 0.05) between concentrations of 10 cfu/mL and 10^6^ cfu/mL and control (no *E. coli*) occurred (Figure 4A,B). However, based on Tukey HSD test, no significant difference was observed between 10 cfu/mL and 10^4^ and 10^5^ cfu/mL (*p* > 0.05). Similar results were also observed at higher concentrations in filtered tap water, with no statistical difference (*p* > 0.05) between 10^4^ and 10^6^ cfu/mL.

**Figure 5:**
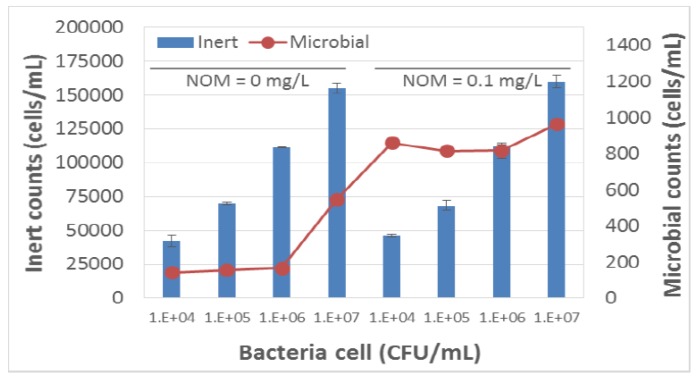
The Sensor B response to *E. coli* spiked in DI water with or without Natural Organic Matter (NOM).

**Figure 6:**
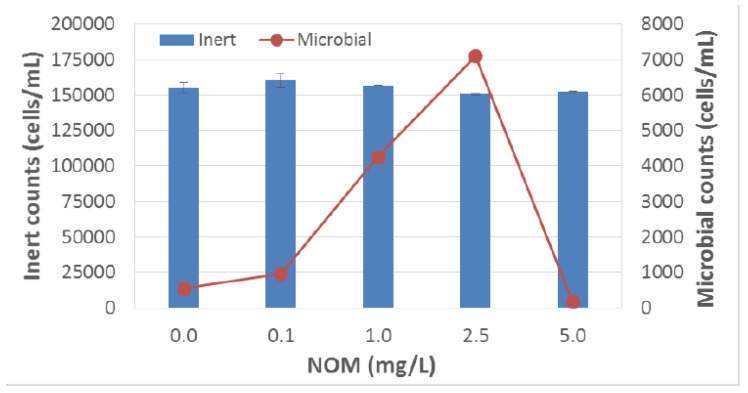
The Sensor B response to *E. coli* spiked (10^7^ cfu/mL) in DI water containing Natural Organic Matter (NOM).

The response of this sensor to bacteria within modelled reclaimed water was also revaluated. Specifically, the bacterial count from secondary effluent obtained from a conventional wastewater treatment plant was evaluated via the sensor and culturable assays. However, viable counts based on fluorescence measured by the sensor did not correlate with culturable counts obtained by plating on R2A media (Figure 5 and Figure 6). We hypothesized that the inability of the sensor to detect fluorescence from NADP and riboflavin in modelled reclaimed water was likely due to interference from natural organic matter (NOM) contained within the water. Therefore, we further evaluated the performance of this sensor in the presence of organic matter.

**Figure 7:**
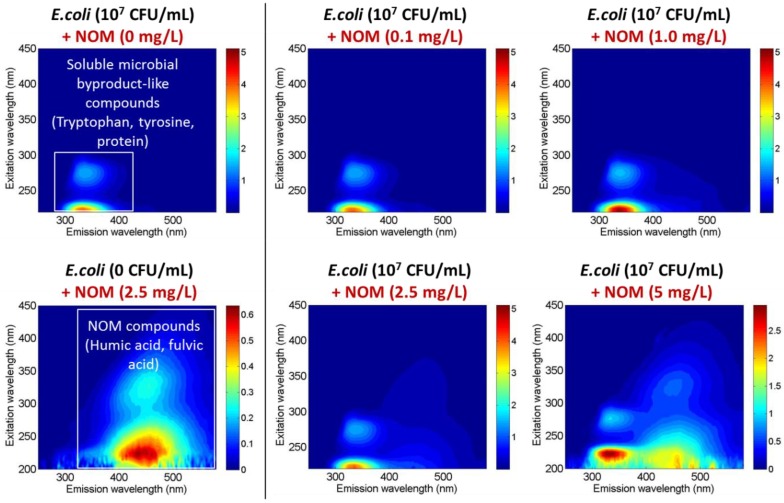
The FL-EEMs of either *E. coli* & Natural Organic Matter (NOM) in DI water.

Specifically, fluorescence excitation emission regions in response to *E. coli* and/or NOM were determined. Figure 7 shows the fluorescence excitation emission (FL-EEM) of *E. coli* and NOM separately and combined, which explains the performance of this sensor in the absence and presence of organic matter in water. The fluorescence Ex/Em regions of biomarkers (NADPH and riboflavin) that are indicators for viable microorganisms overlap with the regions of NOM fluorescence. The presence of organic matter is therefore interfering with the sensor signal for viable microorganisms. Thus, it is clear that the sensor is able to detect the microorganisms only in the absence of organic matter, so it can potentially be applied to finished water (clean or drinking water) but not secondary effluent or reclaimed water.

### 3.3. Real-Time Detection via ATP Production [Sensor C]

Table 1 shows the raw microbial counts in feed waters, and in permeates and brines following RO. RO treatment of the feed waters resulted in a decrease in heterotrophic plate counts (HPC) of 2 orders of magnitude in the effluent from Green Valley and one order of magnitude from the Ina Road feed water. This assay counts from both effluents were similar to HPC culturable counts.

Table 2 shows the raw microbial counts in the various blends of Brine:Permeate representing incremental treatment failure mode. From these data, correlations of the different microbial assays were developed as a function of salt passage. The correlation between the sensor C values and salt passage for the combined waters. These data indicate that the increased total microbial load associated with increased salt passage can be detected in real-time (<1 min) by this assay. Note that the total microbial load expressed as a function of ATP includes both culturable and non-culturable microbes and includes both prokaryotic and eukaryotic activity. Based on these data, the sensor C would appear to have excellent potential as a surrogate parameter for microbial loads in test waters and be useful for process control in treatment trains.

## 4. Conclusions

The detection of microbes in real-time poses unique problems and difficulties that are different from the real-time detection of chemicals. For example, detection of a bacterial cell is not sufficient since the issue of viability must also be addressed. Other issues include potential osmotic shock in pristine waters and the presence of NOM in reclaimed waters. Specifically, the water quality of the test water being monitored determines which real-time assay should be utilized. In low turbidity potable waters, the MALS assay may be suitable [7,8,9,10]. For fluorescence emission, the monitored water must have low NOM content for the assay to function. Thus, the sensor may be most useful for good quality potable waters, or at the back end of an advanced treatment train for reclaimed water. Note also that both sensors do not detect pathogens *per se*. Instead their ability relies on their ability to function as a real-time trigger. Thus, if increased cell counts are detected (deviation from a stable count), this may be indicative of an intrusion event or incremental treatment failure.

In addition to the on-line sensors, indirect detection of microbial loads via ATP production in a batch test also showed value. Although not on-line, the sensor C was fast (<1 min) inexpensive and could be conducted multiple times a day as needed, to provide process control for advanced treatment of reclaimed water or provide security against intrusion events. In addition, this assay provides good information on total microbial load, including non-culturable microbes, and only provides data on viable (or very recently viable) microorganisms. Based on these studies, real-time assays for microorganisms are feasible and would be value added for utilities. That being said, challenges still remain such as the level of sensitivity required to detect even slight incremental treatment failure [10,11].

## Figures and Tables

**Table 1:** Raw microbial counts in waters from Green Valley (GV) and Ina Rd (Ina) WWTPs.

		Sensor C (ME/100 mL)	HPC (Counts/100 mL)
GV ^1^	Feed water	4.4 × 10^7^	3.8 × 10^8^
	Permeate	2.4 × 10^6^	2.6 × 10^6^
	Brine	2.4 × 10^8^	2.2 × 10^8^
Ina ^2^	Feed water	9.2 × 10^7^	2.6 × 10^8^
	Permeate	6.2 × 10^6^	1.0 × 10^7^
	Brine	4.1 × 10^8^	2.9 × 10^8^

^1^ Green Valley WWTP effluent; ^2^ Ina Rd WWTP effluent.

**Table 2:** Raw microbial counts in blends of Brine: Permeate representing incremental failure.

	Target Blending Ratio (%Brine:%Permeate)	Salt Passage (%)	Sensor C (ME/100 mL)	HPC (Counts/100 mL)
GV	0.1	0.2	3.0	2.8
	0.5	2.0	3.1	2.5
	1	4.6	3.0	2.5
	2	9.1	6.9	2.1
Ina	0.1	0.6	5.6	12.5
	0.5	2.3	6.3	14.5
	1	4.7	6.1	16.0
	2	9.3	10.1	18.0
